# A Promoter Collection for Cell‐Targeted Analysis Within the Stomatal Complex

**DOI:** 10.1002/pld3.70045

**Published:** 2025-02-12

**Authors:** Thanh‐Hao Nguyen, Jovaras Krasauskas, Thu Binh‐Anh Nguyen, Azka Noureen, Mark Smedley, John M. Christie, Wendy Harwood, Michael R. Blatt, Penny Hundleby

**Affiliations:** ^1^ Laboratory of Plant Physiology and Biophysics and the Plant Science Group, School of Molecular Biosciences, Bower Building University of Glasgow Glasgow UK; ^2^ John Innes Institute Norwich UK; ^3^ Phytoform, Rothamsted Research Harpenden UK

**Keywords:** guard cell, foliar epidermis, tissue‐specific expression, *Arabidopsis*, *Brassica*, *Hordeum*

## Abstract

Stomatal aperture is driven by changes in turgor of the guard cells that surround the stomatal pore. Epidermal cells immediately surrounding the guard cells are thought to contribute to the kinetics of aperture changes through changes in their turgor that opposes the guard cells and thought their putative roles in solute storage for use by the guard cells. Nonetheless, our knowledge remains fragmentary of surrounding cell mechanics, in large part because the tools and strategies needed to target the surrounding cells independent of the guard cells are limited. Here, we have analyzed sets of promoters for *Arabidopsis*, *Brassica*, and barley that will allow physiological studies of the roles of epidermal cells and also surrounding cells in the case of barley in stomatal behavior. These tissue‐specific promoters offer distinct advantages over widely used, constitutive promoters by enabling precise and targeted gene expression within guard cells and the adjacent epidermal cells. As genetic tools, the promoters will have applications in strategies centered on physiological analyses and differential comparisons following expression targeted between the guard cells and the foliar epidermis as a whole. As such, they are well suited to questions around the mechanics of solute and water flux that will advance an understanding of the stomatal complex in these model species.

## 1 Introduction

Stomata are pores that provide the major route for gaseous exchange between the interior of the leaf and the atmosphere (Farquhar and Sharkey 1982; Willmer and Fricker 1996; Blatt et al. 2022). They open and close to protect against leaf drying while enabling CO_2_ entry into the leaf for photosynthesis (Jezek and Blatt 2017). Stomatal aperture in most plants tracks the immediate demand for CO_2_ by photosynthetic tissues within the leaf, hence opening in the light and closing in the dark or when the light falls (Lawson and Blatt 2014; Matthews and Lawson 2019). Stomata exert a major control on the water and carbon cycles of the world: Their activities have proven vital to global atmospheric modeling and weather prediction for over a quarter of a century (Beljaars et al. 1996) and are key factors behind the crisis in water availability and crop production that is unfolding with global climate change (Unesco 2015). Thus, stomata represent an important target for breeders interested in manipulating crop performance (Lawson and Blatt 2014; Matthews and Lawson 2019; Blatt and Alvim 2022; Horaruang et al. 2022).

It has long been recognized that stomatal aperture is driven by changes in turgor of the guard cells that surround the stomatal pore. Guard cells respond to environmental, hormonal, and other signals by coordinating transport to alter the solute content, volume, and turgor of the guard cell and drive stomatal movements (Jezek and Blatt 2017). At a first glance, transport across the guard cell membrane, primarily of K^+^, Cl^−^, and malate, dominates the changes in stomatal aperture (Raschke 1977; Lawson and Blatt 2014; Jezek and Blatt 2017). However, the cells surrounding the guard cells also play a role, especially in the kinetics of aperture changes.

The epidermal cells adjacent to the guard cells arise during cell divisions that generate the guard cells and are commonly smaller than those distal from the guard cells (Bergmann and Sack 2007). In some species, they are spatially ordered as pairs or rings of so‐called subsidiary cells surrounding the guard cells. However, in all species, these adjacent cells, hereafter referred to simply as surrounding cells, make up the stomatal complex together with the guard cells (Bergmann and Sack 2007; Franks and Farquhar 2007; Lawson and Blatt 2014).

Turgor of these surrounding cells, whether of specialized subsidiary cells or the adjacent epidermal cells, opposes that of the guard cells and can strongly affect stomatal dynamics (Franks and Farquhar 2007; Lawson and Blatt 2014; Jezek et al. 2019). Eliminating this “back pressure” constraint promotes stomatal opening (Edwards, Meidner, and Sheriff 1976; Edwards and Meidner 1979; MacRobbie and Lettau 1980a, 1980b; Bowling 1987; Franks and Farquhar 2007). The surrounding cells are thought also to store solute for use by the guard cells during stomatal opening (Raschke and Fellows 1971; Willmer and Fricker 1996; Franks and Farquhar 2007), which may explain why genetically ablating the surrounding cells greatly slows stomatal opening and closing in *Brachypodium* (Raissig et al. 2017). Certainly, quantitative modeling of stomata, centered on membrane transport, do not adequately reproduce a full range of stomatal kinetics (Chen et al. 2012; Hills et al. 2012; Wang, Hills, and Blatt 2014) unless an accounting for this “back pressure” and solute “shuttling” is included (Jezek et al. 2019; Jezek et al. 2021; Blatt et al. 2022).

Yet, despite their implicit importance for stomatal dynamics, our functional knowledge remains sparse when it comes to surrounding cell mechanics and its coordination with that of the guard cells. A major challenge has been to develop the tools and strategies needed to target the surrounding cells independent of the guard cells, for example, to manipulate their transport and other characteristics. These tools and strategies are vital, especially in efforts to improve stomatal function in crops. Here, we summarize work identifying sets of promoters for *Arabidopsis*, *Brassica*, and barley that allow physiological study of the roles in stomatal behavior of epidermal cells and surrounding cells in the case of barley.

## 2 Results

We screened the *Arabidopsis*, *Brassica*, and barley literature for a range of genes and their promoters that might yield tissue‐specific expression either in guard cells, in foliar epidermal cells, or in both guard cells and epidermal cells. Candidates were also assessed for their potential for expression primarily in vegetative, aerial tissues, their constitutive and developmental independence, and their likely utility across species platforms. Our primary focus was on promoters that express in the mature cell types and might be useful in studies of stomatal mechanics. These considerations led us to select a total of 15 promoters (Table 1), a few of which are known to be functional in at least two of the three plant models of interest (Kelly et al. 2017). The promoter were selected from multiple plant species, from both dicots (*Arabidopsis* and *Solanum tuberosum* ) and monocots ( *Brachypodium distachyon* , *Hordeum vulgare* , *Oryza sativa* , *Triticum aestivum* , and *Zea mays* ). With the exception of the promoters from barley, all other promoters showed the activities in the epidermis (pAtML1, pAtCER6, and pTaGstA + TaWIR1) (Abe, Takahashi, and Komeda 2001; Hooker, Millar, and Kunst 2002; Altpeter et al. 2005; Sullivan et al. 2016), subsidiary cells (pZmCST1) (Wang et al. 2019), or guard cells (Plesch, Ehrhardt, and Mueller‐Roeber 2001; Galbiati et al. 2008; Yang et al. 2008; Zhang et al. 2011; Rusconi et al. 2013; Raissig et al. 2016). We ruled out several other promoters, including the AtPATROL1 promoter (Higaki et al. 2014) that appears associated with cell development or maturation and AtKC1 promoter that is known to express throughout the epidermis (Nieves‐Cordones et al. 2022).

**TABLE 1:** Promoters and their origins. Sequences of *Arabidopsis* promoters were retrieved from the Arabidopsis Information Resource (TAIR, https://www.arabidopsis.org/). Database of Ensembl Plants (https://plants.ensembl.org/) was used to access promoter sequences of *Brachypodium distachyon* , *Hordeum vulgare* , *Oryza sativa* , and *Zea mays* . Promoter sequence of *StKST1* was available with accession number AJ242852 in GenBank database (NCBI, https://www.ncbi.nlm.nih.gov/). The chimeric promoter sequence of *TaGstA1* and *TaWIR1* was obtained from the binary vector pIPKb005 (GenBank, EU161571).

Origin	Promoter	Size (bp)	Position from ATG	Locus/accession no. of downstream gene	Protein encoded by downstream gene	Reported expression patterns	References
*Arabidopsis thaliana*	AtML1	3443	−5032..−1590 (no 5^′^UTR)	AT4G21750	Meristem layer1	Epidermis	Abe, Takahashi, and Komeda (2001); Sullivan et al. (2016)
	AtCER6	1292	−1292..−1	AT1G68530	3‐Ketoacyl‐coa synthase6	Epidermis	Hooker, Millar, and Kunst (2002)
	AtCYP86A2	1263	−1263..−1	AT4G00360	Cytochrome p450 86a2 monooxygenase	Hypocotyl, cotyledon, root vascular system, stigmatic tissues, ovules, and guard cells of real leaves, sepals, anthers, carpels and styles	Galbiati et al. (2008)
	AtEXPA1	1521	−1521..−1	AT1G69530	Expansin1	Guard cell	Zhang et al. (2011)
	AtGC1	971	−971..−1	AT1G22690	GA‐stimulated regulatory protein	Guard cell	Yang et al. (2008)
	AtMYB60	1327	−1346..−20 (part of 5’UTR)	AT1G08810	MYB60 transcription factor	Guard cell	Rusconi et al. (2013)
*Brachypodium distachyon*	BdSCRM2	1659	−1659..−1	BRADI_2g59497v3	Scream2	Throughout the stomatal lineage	Raissig et al. (2016)
*Hordeum vulgare*	HvCER6	2154	−2154..−1	HORVU.MOREX.r3.4HG0395960	3‐Ketoacyl‐coa synthase6	n.d.	Hooker, Millar, and Kunst (2002); Li et al. (2013)
	HvLTP7a2b	2093	−2093..−1	HORVU.MOREX.r3.5HG0464400	Lipid transfer protein 7a2b	Leaf epidermis	Hollenbach et al. (1997)
	HvMYB61	2030	−1527..503 (2 first exons and 2 first introns)	HORVU.MOREX.r3.1HG0018590	MYB61 transcription factor	n.d.	This study
	HvSNAC1	900	−900..−1	HORVU.MOREX.r3.5HG0524540	Stress‐responsive NAC1 transcription factor	n.d.	Al Abdallat et al. (2014); Kurowska and Daszkowska‐Golec (2023)
*Oryza sativa*	OsSAPK10	2005	−2005..−1	Os03g0610900	SnRK2 protein kinase10	Shoot and root	Kobayashi et al. (2004)
*Solanum tuberosum*	StKST1	679	−679..−1	NM_001288546/X79779	Inwardly rectifying K^+^ channel1	Guard cell and flower base	Plesch, Ehrhardt, and Mueller‐Roeber (2001)
*Triticum aestivum*	TaGstA1 fused with TaWIR1 (chimeric)	2554	−2296..−1	XM_044491896/X56012 (*TaGstA1*)	Glutathione‐S‐transferase1	Shoot epidermis and phloem	Altpeter et al. (2005)
			−46..−1 + 50..261	M95500 (*TaWIR1*)	Wheat‐induced resistance1		
*Zea mays*	ZmCST1	2022	−2022..−1	GRMZM2G153358/Zm00001eb288410	Closed stomata1	Subsidiary cell	Wang et al. (2019)

Abbreviation: n.d., not yet determined.

**FIGURE 1:**
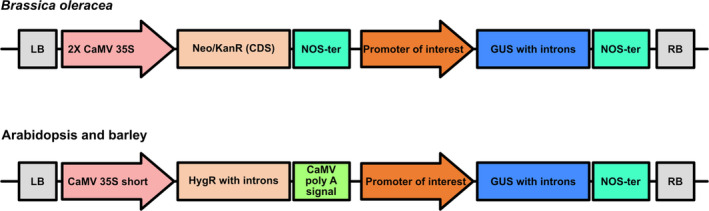
Construct designs for *GUS* expression driven by the promoters of interest. To build the expression vectors, two cassettes, one for *GUS* and one for the selection marker gene, were introduced into the Level 2 vector pAGM8031, between the left border (LB) and right border (RB) sites. For *Brassica oleracea* (above), the kanamycin selection marker was used instead of the hygromycin selection marker that was used for *Arabidopsis* and barley (below).

The promoter sequences were used to generate GoldenGate‐compatible DNA syntheses for ligation in the pUC57 vector. Constructs were assembled (Figure 1) by incorporating within the relevant expression cassette the β‐glucoronidase (*GUS*) gene for expression driven by each promoter and constitutive 35S‐driven kanamycin resistance (*Brassica*) or hygromycin resistance (*Arabidopsis* and barley) for *Agrobacterium* transformation. All cassettes were terminated with NOS terminators except for hygromycin resistance, which ended with CaMV poly(A) signal terminator. The constructs were sequence‐verified before transformation. Stable transformants were generated by floral dip in *Arabidopsis* (Clough and Bent 1998), petiole base transformation in *Brassica* (Hundleby and Chhetry 2020), and immature embryo transformation in barley (Hinchliffe and Harwood 2019). For *Brassica* and barley, only transgenic lines having one or two copies of the transgene were selected for expression pattern analysis.

### 2.1 Arabidopsis

**FIGURE 2:**
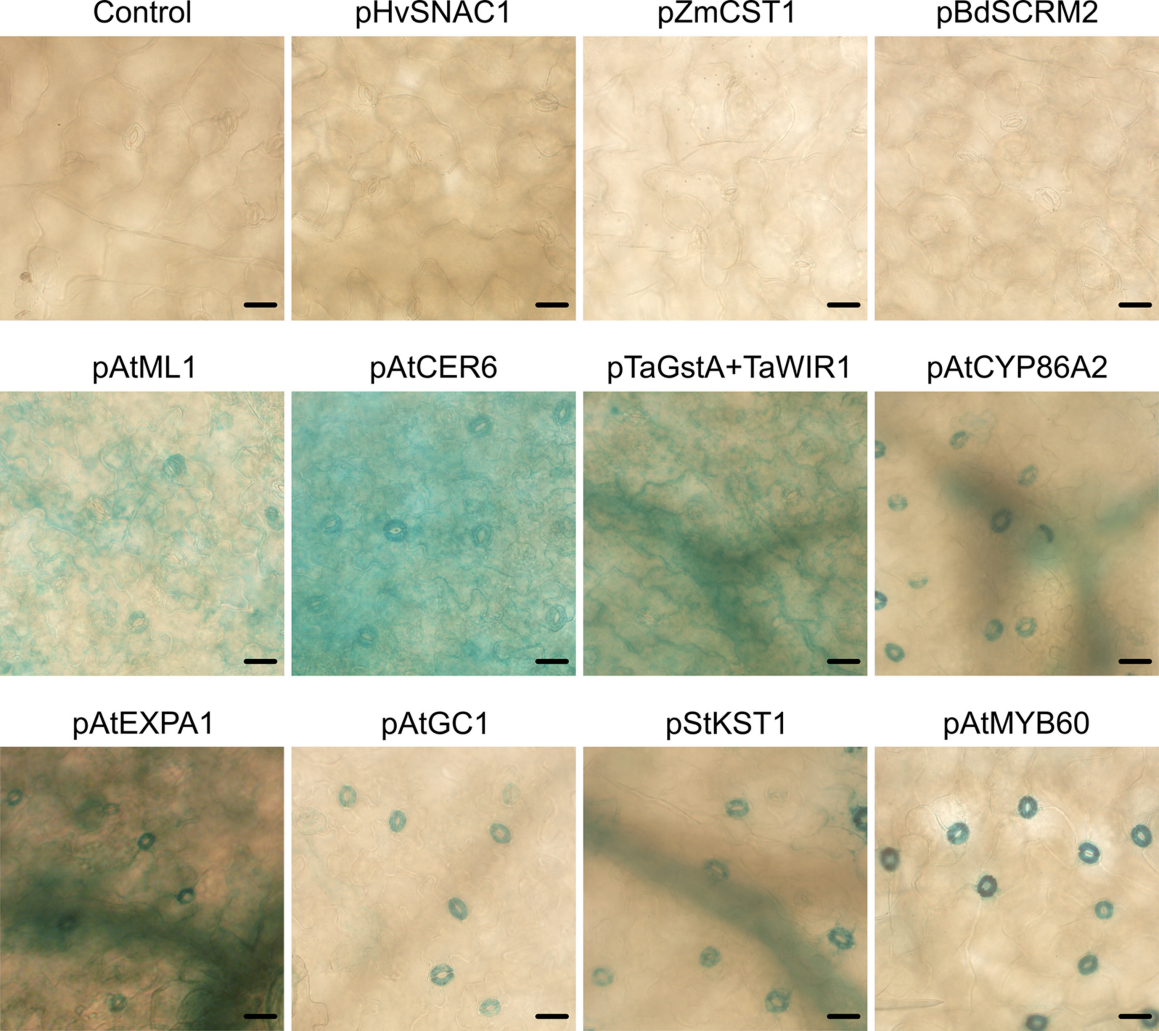
The GUS activity of 11 promoters in *Arabidopsis* leaves. GUS histochemical staining was performed on leaves of transgenic plants carrying the *promoter::GUS* constructs. Each panel representative of expression patterns observed from two or more independent homozygous lines. Leaves were incubated at 37°C overnight in GUS solution supplemented with 2.5 mM ferricyanide and 2.5 mM ferrocyanide. Scale bars, 20 μm.

**FIGURE 3:**
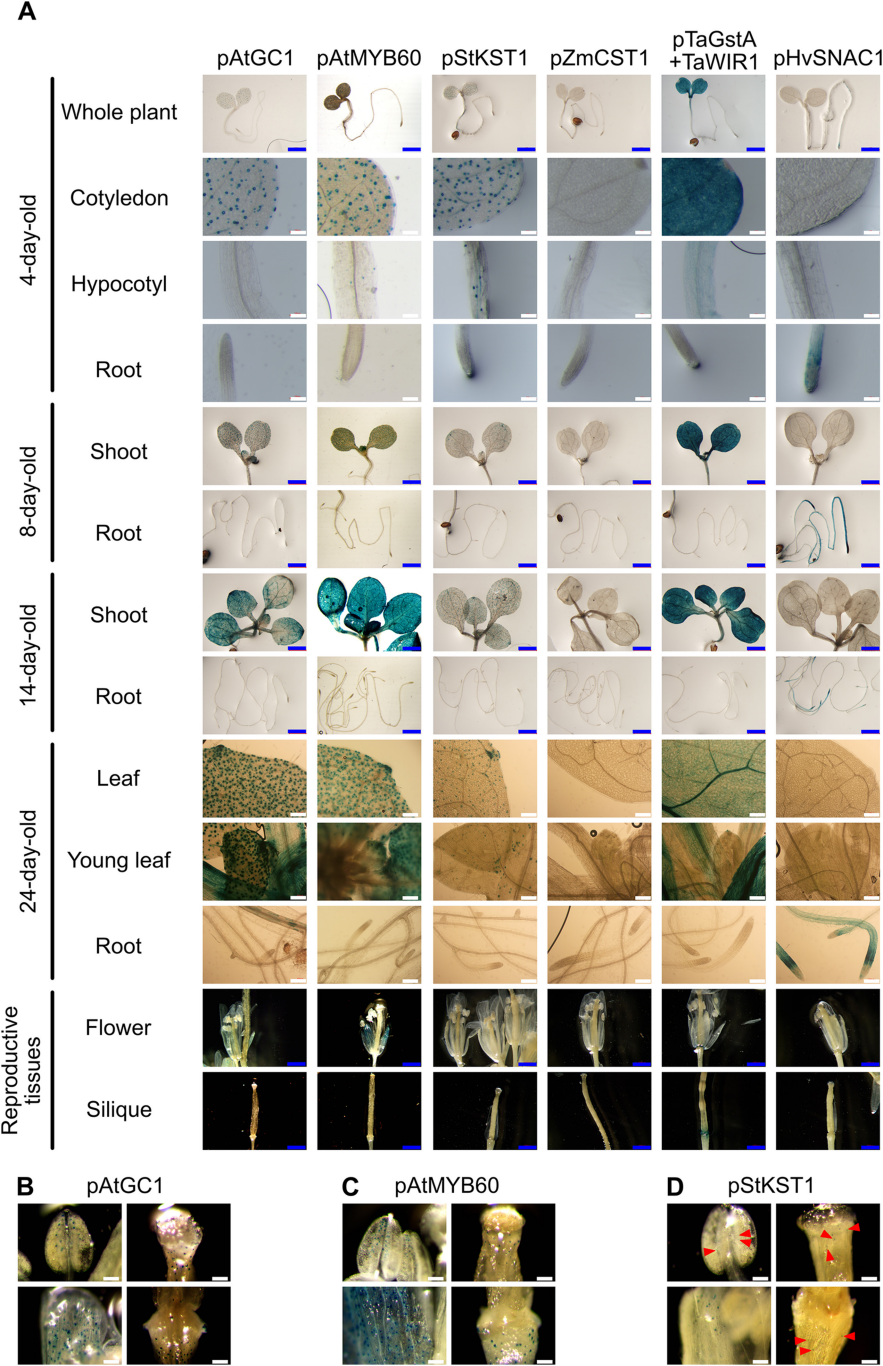
The GUS activity over different developmental stages in *Arabidopsis*. (A) GUS histochemical staining of 4‐, 8‐, 14‐, and 24‐day‐old transgenic plants and the reproductive tissues including flowers and siliques of mature plants. (B–D) Anther, sepal, top, and base of young silique of *pAtGC1::GUS*, *pAtMYB60::GUS*, and *pStKST1::GUS* plants showing GUS activity in guard cells (red arrows indicate guard cells with weak GUS signal). Samples were incubated at 37°C overnight in GUS solution supplemented with 2.5 mM ferricyanide and 2.5 mM ferrocyanide. Scale bars, 100 μm (white) and 1 mm (blue).

Expression of GUS was recovered in Arabidopsis driven by 11 of the promoters (Tables 1 and 2 and Figures 2 and 3). Two of the dicot promoters, pAtGC1 and pAtMYB60, showed primarily a guard cell‐specific pattern of expression, whereas pAtCYP86A2, pStKST1, and pAtEXPA1 also occasionally showed expression in vascular tissues. The pAtMYB60 promoter has been reported to give a low level of expression in other epidermal tissues, including root hairs (Cominelli et al. 2011; Oh et al. 2011). We cannot rule out such expression, either with pAtMYB60 or any other promoter, but note that it is likely to be below the resolution that can be achieved by GUS histochemical analysis. The promoters pAtML1 and pAtCER6 yielded GUS expression in epidermal and guard cells; and pTaGstA‐TaWIR1 showed strong activity throughout the leaf at early stages of development (Figure 3, up to 14 days old), but later the activity was restricted mainly to the epidermis and vascular tissues. We observed strong nonspecific GUS signals in the aerial part at the 14‐day‐old stage when driven by the two promoters pAtGC1 and pAtMYB60. We failed to recover expression driven by the other promoters.

**TABLE 2:** Summary of expression patterns of the investigated promoters. The expression patterns is presented as plant organs (with cell/tissue specific) showing the promoter activity.

Origin	Promoter	*Arabidopsis*	*Brassica*	Barley
*Arabidopsis thaliana*	AtML1	Leaf^a^ (GC, EP)	Leaf^a^ (GC, EP)	Nonactive in leaf^a^
	AtCER6	Leaf^a^ (GC, EP)	Leaf^a^ (GC, EP)	Nonactive in leaf^a^
	AtCYP86A2	Leaf^a^ (GC, VASoc)	Leaf^a^ (GC, EP, VAS)	Nonactive in leaf^a^
	AtEXPA1	Leaf^a^ (GC, EP, VASoc)	Leaf^a^ (GC, EP, VAS)	Nonactive in leaf^a^
	AtGC1	Leaf (GC, EP^b^) Cotyledon (GC) Anther (GC) Sepal (GC) Silique (GC) Receptacle (GC)	Leaf^a^ (GC)	Nonactive in leaf^a^
	AtMYB60	Leaf (GC) All aerial parts^b^ Cotyledon (GC) Hypocotyl (GC) Anther (GC) Sepal (GC) Silique (GC) Receptacle (GC)	Leaf^a^ (GC)	Nonactive in leaf^a^
*Brachypodium distachyon*	BdSCRM2	Nonactive in leaf^a^	Nonactive in leaf^a^	Nonactive in leaf^a^
*Hordeum vulgare*	HvCER6	n/a	n/a	Leaf^a^ (GC, EPw, VAS)
	HvLTP7a2b	n/a	n/a	Leaf^a^ (GCw, EPw, VASw)
	HvMYB61	n/a	n/a	Nonactive in leaf
	HvSNAC1	Nonactive in all aerial parts Root (strongest in the elongation zone)	Nonactive in leaf^a^	Leaf^a^ (GC, EP, VAS)
*Oryza sativa*	OsSAPK10	n/a	n/a	Nonactive in leaf^a^
*Solanum tuberosum*	StKST1	Leaf (GC, VAS oc) Cotyledon (GC) Hypocotyl (GC) Anther (GC) Sepal (GC) Silique (GC) Receptacle (GC)	Leaf^a^ (GC)	Leaf^a^ (GCw, EPw, VAS)
*Triticum aestivum*	TaGstA1 fused with TaWIR1 (chimeric)	Leaf (GC, EP, VAS) Cotyledon Hypocotyl Junction between silique and receptacle Nonactive in root	Leaf^a^ (GC, EP)	Leaf^a^ (GC, EP, VAS)
*Zea mays*	ZmCST1	Nonactive in all plant off different developmental stages	Nonactive in leaf^a^	Leaf^a^ (SC)

Abbreviations: EP, epidermal cell; GC, guard cell; oc, occasionally; SC, subsidiary cell; VAS, vascular tissues; w, weak expression level.

^a^ Expression patterns analysis was performed only in leaf.

^b^ Specific expression pattern(s) only observed at 14‐day‐old stage.

In addition to post‐seedling vegetative expression, three dicot promoters, such as pAtGC1, pAtMYB60, and pStKST1, and three monocot promoters, such as pHvSNAC1, pZmCST1, and pTaGstA + TaWIR1 (Al Abdallat et al. 2014; Kurowska and Daszkowska‐Golec 2023), were evaluated across developmental stages in homozygous lines. We examined expression in 4‐, 8‐, and 14‐day‐old seedlings, in mature leaves, in flowers, and during seed development. The activity of the three dicot promoters in 4‐day‐old seedlings was specific to guard cells, but though pAtGC1 was active in guard cells of the cotyledon, pStKST1 and pAtMYB60 activity extended to guard cells of the hypocotyl (Figure 3A). The pHvSNAC1 promoter showed activity only in roots, whereas pTaGstA + TaWIR1 yielded a strong GUS signal in aerial parts only, notably also in vascular tissues. The subsidiary cell‐specific promoter pZmCST1, derived from the monocot, was not functional in *Arabidopsis*. Otherwise, these promoters gave largely stable GUS signals throughout development, with the exception of pAtMYB60 and pHvSNAC1. pAtMYB60 showed strong expression in all the 14‐day‐old aerial part, whereas pHvSNAC1 showed a decline in the GUS signal in older tissues and was restricted in the elongation zone of the 24‐day‐old root (Figure 3A). A GUS signal was not detected in reproductive tissues when driven by the pHvSNAC1 and pZmCST1 promoters but was evident in receptacles when driven by pTaGstA + TaWIR1 (Figure 3A) and in guard cells of sepals, anthers, siliques, and receptacles when driven by pAtGC1, pAtMYB60, and pStKST1 (Figure 3B–D). We found the activity of pStKST1 was generally lower in reproductive organs compared to that of pAtGC1 and pAtMYB60 (Figure 3). Although all three promoters—pAtMYB60, pAtGC1, and pStKST1—were found active in guard cells of anthers, the frequency of anthers with GUS signals in guard cells was low when driven by pAtGC1 and pStKST1, whereas expression was common when driven by pAtMYB60.

### 2.2 Brassica

**FIGURE 4:**
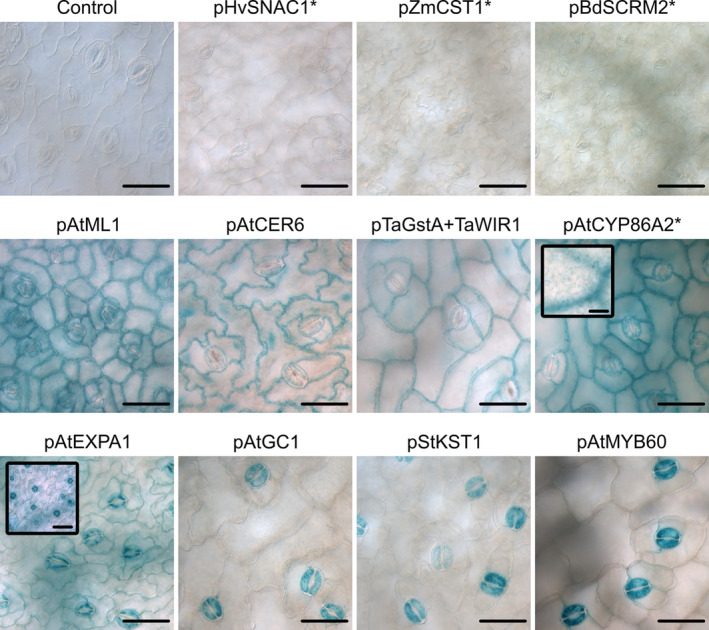
The GUS activity of 11 promoters in *Brassica oleracea* leaf. GUS histochemical staining was performed on leaves of transgenic plants carrying the *promoter::GUS* constructs. Each panel representative of expression patterns observed from two or more independent homozygous lines. Small framed figures at the top left corner of pAtCYP86A2 and pAtEXPA1 panels are additional figures allowing better observation of GUS signal in the vascular tissues. Leaves were incubated at 37°C overnight in GUS solution supplemented with either 2 mM or 1 mM (*) ferricyanide/ferrocyanide. Scale bars, 50 μm.

*Brassica oleraceae* is a cruciferous species and, like *Arabidopsis*, is a member of the Brassicaceae family. As expected, most of the promoters active in *Arabidopsis* were also found to give GUS expression in this crop species (see Tables 1 and 2). The epidermal promoters—pAtML1, pAtCER6, and pTaGstA + TaWIR1—all gave expression in both epidermal and guard cells. Similarly, the pAtGC1, pStKST, and pAtMYB60 promoters all showed strong activity that was largely confined to the guard cells as in *Arabidopsis* (Figure 4). We failed to recover expression in *Brassica* when driven by pZmCST1, pHvSNAC1, and pBdSCRM2.

However, differences in the expression patterns were observed in the case of pAtCYP86A2 and pAtEXPA1. Whereas in *Arabidopsis* leaf epidermis, these two promoters were specific to the guard cells, in *Brassica*, they were found active also in epidermal cells (Figure 4). pAtCYP86A2 showed activity that was similar between guard cells and the adjacent epidermal cells in terms of GUS signal intensity. Such activity is similar to that of pAtML1, pAtCER6, and pTaGstA + TaWIR1. Although also active in both guard cells and epidermal cells, the pAtEXPA1 yielded a much stronger GUS signal in the guard cells than the neighboring cells.

Promoter activities in *Brassica* also mirrored the expression patterns of *Arabidopsis* beyond the epidermal and guard cells. We observed some scarce GUS signal in vascular tissues when driven by pAtEXPA1 and pAtCYP86A2. However, we again observed a difference in the expression patterns between *Arabidopsis* and *Brassica*, as in the case of pStKST1. Although this promoter is active non‐uniformly in *Arabidopsis* vascular tissues (Figures 2 and 3), we did not observe any GUS signal in vascular tissues of the transgenic *Brassica* lines.

### 2.3 Barley

**FIGURE 5:**
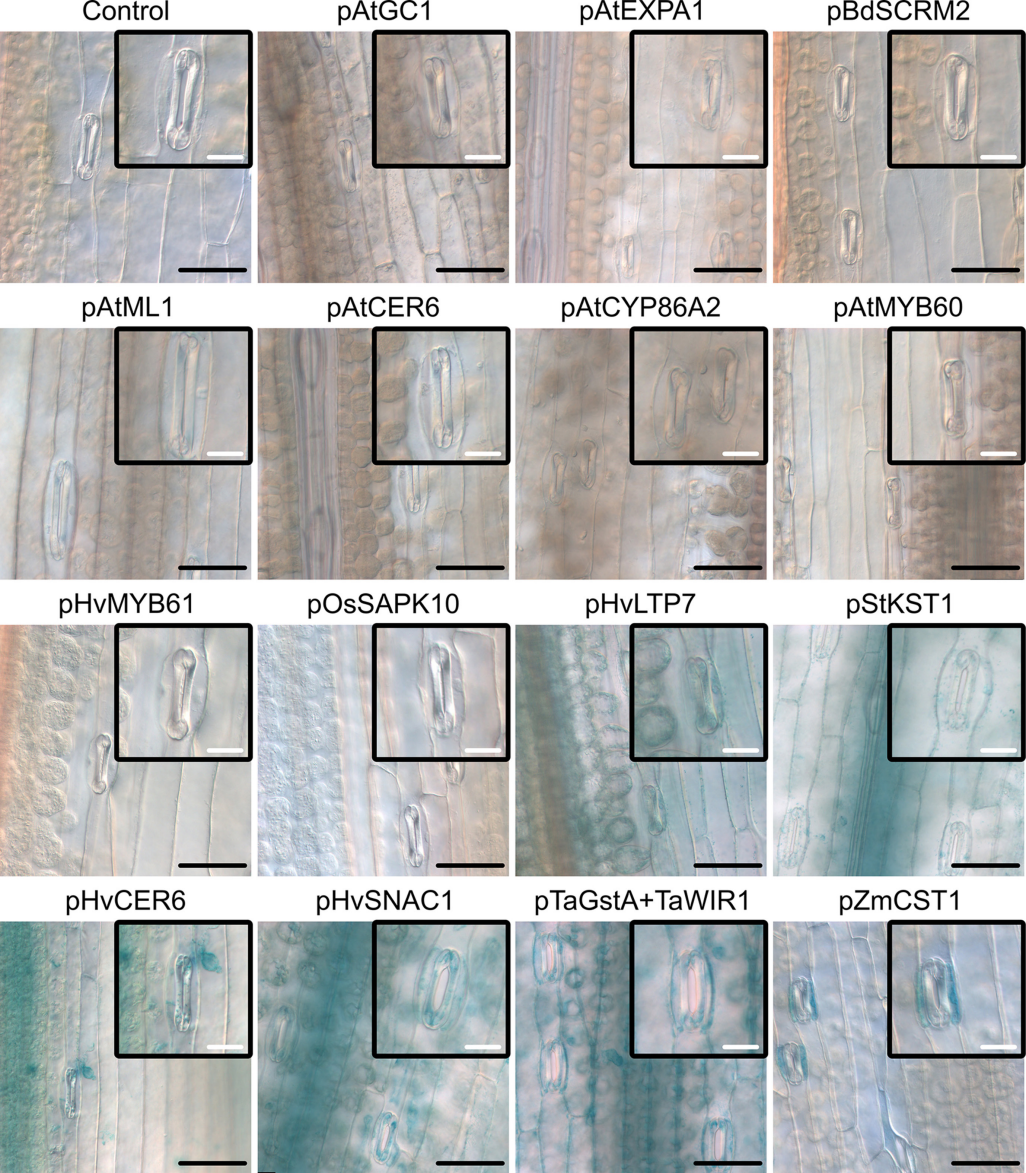
The GUS activity of 15 promoters in barley leaf. GUS histochemical staining was performed on leaves of transgenic plants carrying the *promoter::GUS* constructs. Each panel representative of expression patterns observed from two or more independent homozygous lines. The magnification of a stomatal complex was shown at the top right corner of each panel. Leaves were incubated at 37°C overnight in GUS solution supplemented with 1 mM ferricyanide/ferrocyanide. Scale bars, 20 μm (white) and 60 μm (black).

For barley, the list of investigated promoters was extended to 15 promoters with the addition of three barley promoters (pHvCER6, pHvLTP7, and pHvMYB61) and a rice promoter (pOsSAPK10) (Kobayashi et al. 2004). In barley, a monocot plant, the promoter activities were completely different from that observed in *Arabidopsis* and *Brassica*. The two promoters that were inactive in *Arabidopsis* and *Brassica*, pHvSNAC1 and pZmKST1, drove GUS expression in different cell types in barley leaf. In contrast, most of the promoters active in the two dicot species failed to generate any GUS signal in barley leaf (see Tables 1 and 2 and Figures 2, 4, and 5).

The pBdSCRM2 promoter remained inactive in all three species while the pStKST1 yielded GUS expression, although the expression pattern was different in barley. This potato promoter, which showed expression specific to guard cells in *Arabidopsis* and *Brassica*, was active in all barley epidermal cells including guard cells and in the vascular tissues with a stronger GUS signal observed in the vascular tissues. The same expression pattern in barley leaves was also obtained when driven by the four promoters: pTaGstA + TaWIR1, pHvLTP7, pHvCER6, and pHvSNAC1 (Figure 5). The maize promoter, pZmCST1, was found specifically active in barley subsidiary cells (Figure 5) as was also found in maize (Wang et al. 2019). Among the additional promoters, the activity of the two promoters, pHvMYB61 and pOsSAPK10, could not be detected by GUS histochemical assay (Figure 5).

## 3 Discussion

Although stomatal aperture is driven by changes in turgor of the guard cells, the surrounding epidermal cells also make quantitative contributions, both in the dynamic ranges of apertures achieved (Edwards, Meidner, and Sheriff 1976; Edwards and Meidner 1979; MacRobbie and Lettau 1980a, 1980b) and in the kinetics of opening and closing (Franks and Farquhar 2007; Lawson and Blatt 2014; Jezek et al. 2019; Jezek et al. 2021; Blatt et al. 2022). Yet, with few exceptions (Raissig et al. 2017; Nieves‐Cordones et al. 2022; Cheng and Raissig 2023), there has been little progress toward resolving the mechanics of these contributions, in part for lack of promoters that would enable comparative physiological analysis of contributions from cells in the surrounding epidermal layer.

We have targeted a selection of epidermally associated promoters, screening these for their potential as tools to differentially manipulate gene expression between guard cells and the cells that surround them. Our 15 promoters (Table 1) derive from both monocots and dicots and include three that were either proposed or used previously to drive gene expression in guard cells. The results demonstrate a selection of promoters that are available for guard cell‐specific and epidermal expression in *Arabidopsis* and *Brassica* and for expression in the surrounding cells of barley. By contrast, promoters that target expression to the epidermal cells also showed significant expression in the guard cells of these species. These characteristics do not rule out comparative and physiological studies that rely on selective expression in the guard cells and in their surrounding cells; however, they imply that such studies are likely to require differential approaches that recognize the combined expression across both cell types when using epidermally targeted promoters.

Of the guard cell‐specific promoters, we observed strong and selective expression driven by pAtMYB60, pStKST1, and pAtGC1, all of which have previously proven useful in targeting guard cells in several dicotyledonous species (Cominelli et al. 2005; Yang et al. 2008; Rusconi et al. 2013; Wang et al. 2014; Kelly et al. 2017; Toh et al. 2021). We also observed comparable expression with pAtEXPA1 (Zhang et al. 2011). The promoters pAtMYB60, pAtGC1, and pAtEXPA1 derive from *Arabidopsis*, but pKST1 derives from potato. All four are promoters of genes that are strongly expressed in *Arabidopsis* guard cells, and three of these—pAtMYB60, pAtGC1, and pStKST1—were equally effective in *Brassica* (Figures 2 and 4). In barley where the stomatal complex consists of a pair of thin dumbbell‐shaped guard cells flanked by a pair of subsidiary cells, the *Z. mays* promoter pZmCST1 was most effective as a subsidiary cell–selective promoter.

Among the remaining promoters, we recovered strong GUS expression in the epidermal cell layer with pAtCER6 and, especially, with pAtML1 and pTaGstA + TaWIR1 in both *Arabidopsis* and *Brassica*. In *Brassica*, a similar expression pattern was obtained when the GUS expression was driven by pAtCYP86A2 and pAtEXPA1. Analogous results for barley were evident with GUS expression driven by pHvSNAC1, pHvLTP7, pHvCER6, pTaGstA + TaWIR1, and pStKST1. In each case, GUS expression was evident both in the epidermal pavement and in the guard cells. These results are consistent with the broader nature of the corresponding gene functions. In *Arabidopsis*, *ATML1* expresses a homeodomain‐containing transcription factor specific to the L1 layer (Lu et al. 1996; Abe, Takahashi, and Komeda 2001), *AtCER6* expresses a condensing protein in surface wax production (Hooker, Millar, and Kunst 2002), and the *TaGstA1* gene product is a glutathione S‐transferase active in cellular redox control of the leaf epidermis (Altpeter et al. 2005). *HvLTP7* expresses a general housekeeping lipid transferase in barley (Hollenbach et al. 1997), and *HvCER6* is a cuticle‐associated gene (Li et al. 2013).

A few of the promoters were effective both in the dicotyledonous *Arabidopsis* and *Brassica* and in the monocotyledonous barley. Of these, most notable was pTaGstA + TaWIR1, which showed strong GUS expression across the epidermal cell layer in all three species (Figures 2, 4, and 5). We also observed GUS activity in all three species driven by the pStKST1 promoter; however, whereas this promoter is normally guard cell‐specific in *Arabidopsis* and similarly in *Brassica* (Figures 2 and 4), it yielded a signal throughout the epidermal cell layer, including the guard cells, when introduced into barley (Figure 5). These findings contrast with a previous report suggesting that expression was limited to barley guard cells (Kelly et al. 2017).

In conclusion, we have identified a selection of promoters with the potential for use in tissue‐specific expression and analysis of the physiological interactions thought to take place within the stomatal complex. These promoters offer the means to engineer and manipulate stomatal function in *Arabidopsis*, *Brassica*, and barley, and to advance our understanding of stomatal interactions with the environment. As genetic tools, they enable strategies in physiological analyses that rely on differential comparisons following expression targeted between the guard cells and the foliar epidermis as a whole. Such strategies are well suited to questions centered on the mechanics of solute and water flux between guard cells and their surrounding cells and therefore should help advance an understanding of the stomatal complex in these model species.

## 4 Materials and Methods

### 4.1 Plant Growth Conditions

*B. oleracea* genotype AG DH1012, used in this study, is a spring type and self‐compatible, doubled haploid genotype derived from the crossing between *B. oleracea* ssp. *alboglabra* (A12DHd) and *B. oleracea* ssp. *italica* (Green Duke GDDH33) mapping population. Tissue culture of *B. oleracea* was maintained under long‐day condition (16:8 h light/dark, 40 μmol m^−2^ s^−1^ PAR, 22 °C). Transgenic lines generated through tissue culture were transferred to soil and grown under shade within a propagator for 1 week, ensuring adaptation to reduced humidity and increased light intensity. In the glasshouse, plants were grown under long‐day condition (16 h of natural light with a supplement of 200 μmol m^−2^ s^−1^, day/night temperature, 18:12°C ± 2°C). Plants were fertilized weekly with N:P:K at a 2:1:1 ratio.

Tissue culture of barley ( *H. vulgare* cv. Golden Promise) was maintained under long‐day conditions from selection (16:8 h light/dark, 140 μmol m^−2^ s^−1^ PAR, 24°C). Transgenic lines generated through aseptic culture were transferred to cereal mix. The mix also contained a slow‐release fertilizer (Osmocote used at the manufacturer‐recommended concentration). Plants grown from seed were transplanted into 12‐cm pots once they reached a height of 20 cm and were grown in controlled environment rooms under 16:8 h light/dark, 500 μmol m^−2^ s^−1^ PAR (metal halide lamps supplemented with tungsten bulbs), 15:12°C ± 2°C).

Arabidopsis was grown under short day condition (9 h light/15 h dark, 150 μmol m^−2^ s^−1^ PAR, 60% relative humidity). Transgenic plants were screened on 0.5x Murashige & Skoog (MS) plates (pH 5.8, 0.8% w/v agar) containing appropriate antibiotics and transferred to soil pot for seed production after selection.

### 4.2 Vector Construction

Fifteen promoters across monocot and dicot plants were selected based on their tissue‐, cell type‐specific, and/or stress‐inducible activity (see also Table 1). Sequences of promoters were commercially synthesized and introduced into pUC‐GW‐Kan vector at its multicloning sites (Genewiz). Each synthesized promoter was flanked by recognition sites of the Type IIS enzyme *Bsa*I to facilitate golden gate cloning in the following steps (Engler et al. 2014). Recognition sequences of *Bsa*I were designed to situate at the very ends of the promoter sequences, outside of the restriction sites, ensuring the sites were eliminated in the final constructs after digestion and ligation. The promoter components were designed to have standardized four‐base flanking overhangs after cleavage. Three‐component Level 0 vectors each carrying promoter (pUC57), *GUS* gene (pICH75111), and NOS terminator (pICH41421) and empty Level 1 acceptor vector (pICH47742) were digested by *Bsa*I and ligated directionally, based on the customized four base flanking overhangs to obtain complete *promoter:GUS:NOS* cassette in the Level 1 vector pICH47742.

To build the final Level 2 plant expression vector, the two Level 1 vectors each carrying hygromycin selection (pICSL11059) and *promoter:GUS:NOS* cassettes (in pICH47742) and the empty Level 2 vector (pAGM8031) were digested by *Bpi*I and assembled directionally based on the four‐base flanking overhangs. Also, in this reaction, a small fragment digested from pICH41744 (by BbsI) acted as the linker between *promoter:GUS:NOS* cassette and the Level 2 vector pAGM8031. These plant expression vectors were then used for *H. vulgare* and *Arabidopsis* transformation. Those for *B. oleracea* transformation were constructed using a similar approach but designed to have kanamycin selection marker instead; thus, the Level 1 vector harboring kanamycin selection cassette (pICSL11055) was used alternatively when building the *B. oleracea* Level 2 expression vector. To ensure the success of golden gate cloning approach, the native recognition sites of either or both Type IIS enzymes, exist within sequences of six out of 15 selected promoters (AtCER6, AtCYP86A, AtEXPA1, BdSCRM2, TaGstA + TaWIR1, and ZmCST1), were eliminated by introducing silent point mutations through gene synthesis. These mutations were confirmed not situate in any *cis*‐element motifs, thus having no effect on transcription factor binding and promoter activity. All vectors and modules used were sourced from the Sainsbury Laboratory (https://synbio.tsl.ac.uk/) and are available from Addgene (https://www.addgene.org/). Plant transformation vectors containing promoter sequences constructed in this study are listed in Table S1.

### 4.3 Stable Plant Transformation and Transgenic Line Selection

*Agrobacterium tumefaciens* strain AGL1 was transformed with confirmed vectors and used for Arabidopsis, *B. oleracea* , and *H. vulgare* transformation.

*Arabidopsis thaliana* Col‐0 grown under long day condition (16:8 h light/dark, 150 μmol m^−2^s^−1^PAR, 60% relative humidity) was used for *Agrobacterium*‐mediated transformation by floral dip method (Clough and Bent 1998). T_1_ seeds were sown and screened on MS plates (above) with 25 μg mL^−1^ hygromycin. Hygromycin‐resistant T_1_ seedlings with elongated hypocotyls and emerging green leaves were selected and transferred to soil followed by preliminary GUS histochemical analysis. For each *promoter::GUS* construct, 6–10 independent T_1_ lines were analyzed and further kept for T_2_ seed production. Independent lines at the T_2_ generation were used for promoter activity analysis in mature leaf and in those under different developmental stages.

*B. oleracea* was transformed using cotyledonary petioles isolated from 4‐day‐old seedlings co‐cultivated with *A. tumefaciens* strain AGL1 harboring the appropriate plasmid to be evaluated (Hundleby and Chhetry 2020). Putative transgenic shoots regenerated from callus at the petiole base after 4 weeks were isolated and transferred to root induction medium containing 25 μg mL^−1^ kanamycin. The presence of each transgene was confirmed by GUS assay and qRT‐PCR (to determine T‐DNA copy number using the nptII selectable marker gene). Transgenic T_0_ plants were transferred to soil and kept for T_0_ seed production.

*H. vulgare* transformation was performed by co‐cultivation of immature embryos isolated from sterilized seeds with *Agrobacterium* (Hinchliffe and Harwood 2019). Co‐cultivated immature embryos were transferred to callus inducing medium containing 30 μg mL^−1^ hygromycin. Samples from calli derived from immature embryos were analyzed for *GUS* expression and positive calli further regenerated for shoots and roots. Transgenic T_0_ plantlets were confirmed by GUS staining and qRT‐PCR analysis for transgene copy number and were transferred to cereal mix and grown to maturity for T_1_ seed production.

### 4.4 GUS Histochemical Analysis

For barley and *Brassica*, 1 cm^2^ of T_0_ and T_1_ leaf tissues were collected for GUS expression analysis. For *Arabidopsis*, GUS analysis was done with T_2_ plants, and for some promoters including pAtGC1, pAtMYB60, pHvSNAC1, pStKST1, pTaGstA + TaWIR1, and pZmCST1, GUS expression analysis was intensively analyzed at different developmental stages, including 4‐, 8‐, 14‐, and 24‐day‐old, 5‐week‐old, and flowering. For early developmental stages, T_2_ plants grown on 0.5× MS medium containing hygromycin was used for GUS staining. The plant density was maintained at 10 plants/plate, allowing enough space and nutrient for plants to grow overtime. For 5‐week‐old and flowering stages, 7‐day‐old plants grown in plates were transplanted to soil pots, and leaf tissue was collected for GUS staining. Throughout experiments plants were grown in short‐day condition (9 h light/15 h dark, 150 μmol m^−2^ s^−1^ light intensity, 60% humidity maintained for soil‐growing plants). For all plant species, 2–3 young fully expanded leaves per plant, and 2 plants for each independent line were used for GUS staining.

Harvested samples either leaf or whole plant tissue were rinsed in cold methanol for 30 min followed by sodium phosphate buffer wash. Leaf tissue was subsequently vacuum‐infiltrated with X‐Gluc solution (1 mg mL^−1^ of 5‐bromo‐4‐chloro‐3‐indolyl‐D‐glucuronic acid, 1–2.5 mM K_4_Fe(CN)_6_ and K_3_Fe(CN)_6_, 50 mM sodium phosphate buffer pH 7, and 0.05% Triton X‐100) and incubated overnight at 37°C (Truernit et al. 2008). On the following day, the X‐Gluc solution was removed, and samples were dehydrated in ethanol with increasing concentration from 70% to 100%, concurrently removing chlorophyll. Prior to imaging, samples were treated in a chloral hydrate solution (4 g chloral hydrate, 1 mL glycerol, and 2 mL water). Sample were imaged under Zeiss Stemi 305 Stereo and A1 microscopes (Carl Zeiss, Germany).

### 4.5 Quantitative Real‐Time PCR

To identify transgene copy number, qRT‐PCR was performed using gDNA of transgenic plants as templates, DNA‐specific TaqMan probes labeled with either FAM (blue) or VIC (green) reporter dye, and primers specific to the selection marker and endogenous reference genes. *GLABRA2* (*GL2,* EU826523.1) and *CONSTANS‐like gene2* (*CO2*, AF490469.1) were chosen as they are endogenous single‐copy reference genes of *Brassica* and barley (Griffiths et al. 2003; Chai et al. 2010). The reference gene amplicons were detected using VIC‐labeled probes, whereas the transgene amplicons (*NptII*/*Hyg*) were detected with FAM‐labeled probes. Sequences of primers and TaqMan probes are provided in Table S2.

Each qRT‐PCR reaction had a total volume of 25 μL, which included 1 μL standard gDNA (5‐50 ng), 1 μL oligo primer mix (5 μM each forward and reverse primer of selection marker gene and 2.5 μM each primer for endogenous gene), 1 μL probe mix (5 and 2.5 μM for selection marker and endogenous genes), 12.5 μL of 2x ROX master mix, and remaining volume of DNA/RNA‐ and nuclease‐free water (ABsolute QPCR Mix, AB1139, Thermo Fisher Scientific). The thermal profile started at 95°C for 15 min, followed by 40 cycles of 95°C for 15 s and 60°C for 1 min (Bio‐Rad C1000). Ct values were used to calculate transgene/reference ratios (2^−ΔCt^). In order to determine the copy number of transgene in *Brassica* and barley samples, the resulting transgene/reference ratios were normalized against those of a known positive control containing one copy of the transgene (2^−ΔΔCt^).

## Author Contributions

Michael R. Blatt, Wendy Harwood, John M. Christie, and Penny Hundleby conceived the project; Jovaras Krasauskas, Mark Smedley, and Thanh‐Hao Nguyen designed constructs; Thanh‐Hao Nguyen, Thu Binh‐Anh Nguyen, Jovaras Krasauskas, Azka Noureen, and Mark Smedley developed the GUS‐promoter lines; Thanh‐Hao Nguyen, Thu Binh‐Anh Nguyen, Jovaras Krasauskas, and Azka Noureen analyzed the lines; Thanh‐Hao Nguyen and Azka Noureen assembled the data and images; Michael R. Blatt wrote the manuscript with Thanh‐Hao Nguyen and Thu Binh‐Anh Nguyen; all authors contributed to and approved the manuscript.

## Ethics Statement

This work made no use of either human or animal materials. There are no ethical approvals required.

## Conflicts of Interest

The authors declare no conflicts of interest.

## Supporting information


**Data S1** Supporting Information.

**Table S1** Promoter constructs and Addgene reference numbers.
**Table S2.** Primer sequences used in this study.
