# Supplementary material for: A Promoter Collection for Cell‐Targeted Analysis Within the Stomatal Complex
Source: Plant Direct. 2025 Feb 12;9(2):e70045. doi: 10.1002/pld3.70045 (PMC11815712; doi:10.1002/pld3.70045)
Supplement: Supplementary file 2 — Table S1 Promoter constructs and Addgene reference numbers. Table S2. Primer sequences used in this study. [file PLD3-9-e70045-s002.docx]

| **Promoters Evaluated in** | **Construct ID_with Descriptions** | **Addgene Code** |
| --- | --- | --- |
| Barley | 35S::HPT: pCER6#2::GUS_introns | 215200 |
|  | 35S::HPT: pLTP7::GUS_introns | 215199 |
|  | 35S::HPT: pMYB61::GUS_introns | 215198 |
|  | 35S::HPT: pATML::GUS_introns | 215197 |
|  | 35S::HPT: pCER6::GUS_introns | 215196 |
|  | 35S::HPT: pGC1::GUS_introns | 215195 |
|  | 35S::HPT: pEXPA1::GUS_introns | 215194 |
|  | 35S::HPT: pCYP86A2::GUS_introns | 215193 |
|  | 35S::HPT: pCST::GUS_introns | 215192 |
|  | 35S::HPT: pCSRM2C::GUS_introns | 215191 |
|  | 35S::HPT: pGstA+WIR1a::GUS_introns | 215190 |
|  | 35S::HPT: pKST::GUS _introns | 215189 |
|  | 35S::HPT: pMYB60::GUS_introns | 215188 |
|  | 35S::HPT: pSNAC::GUS_introns | 215186 |
|  | 35S::HPT: pCYP86A2::GUS_introns | 215185 |
| **Brassica oleracea** | **Construct ID_with Descriptions** | **Addgene Code** |
|  | 2X35S::NPTII: pATML::GUS_introns | 215183 |
|  | 2X35S::NPTII: pCER6::GUS_introns | 215182 |
|  | 2X35S::NPTII: pGC1::GUS_introns | 215181 |
|  | 2X35S::NPTII: pEXPA1::GUS_introns | 215180 |
|  | 2X35S::NPTII:pCYP86A2::GUS_introns | 215179 |
|  | 2X35S::NPTII: pCST::GUS_introns | 215178 |
|  | 2X35S::NPTII: pCSRM2C::GUS_introns | 215177 |
|  | 2X35S::NPTII: pGstA1+WIRa::GUS_introns | 215176 |
|  | 2X35S::NPTII: pKST::GUS_introns | 215175 |
|  | 2X35S::NPTII: pSNAC1::GUS_introns | 216215 |
|  | 2X35S::NPTII: pMYB60::GUS_introns | 216214 |

**Supplementary Table 1. Promoter constructs and Addgene refeerence numbers.**

**Supplementary Table 2. Primer sequences used in this study**

| **Primers_ID** | **Sequence (5’🡪3’)** |
| --- | --- |
| NptII_F_B.o | GTGGAGAGGCTATTCGGCTAT |
| NptII_R_B.o | TCGGTCTTGACAAAAAGAACC |
| GL2_ F_B.o | TGGACAAGACATGAGGGTTTC |
| GL2_R_B. o | AACAGGTAACCAGAGGGAGGA |
| Hyg_F_H.v | GGATTTCGGCTCCAACAATG |
| Hyg_R_H.v | TATTGGGAATCCCCGAACATC |
| Col_F_H.v | TGCTAACCGTGTGGCATCAC |
| Col_F_H.v | GGTACATAGTGCTGCTGCATCTG |
| **Probes_ID** | **Sequence (5’🡪3’)** |
| NptII_probe | FAM-CTGCTCTGATGCCGCCGTGTT-TAMRA |
| GL2_probe | VIC-AGCCTACCGGAGTCATAGTTTGCGCTT-TAMRA |
| Hyg_probe | Fam-CAGCGGTCATTGACTGGAGCGAGG-Tamra |
| COL2_probe | VIC-CATGAGCGTGTGCGTGTCTGCG-TAMRA |
