# Supplementary material for: A Promoter Collection for Cell‐Targeted Analysis Within the Stomatal Complex
Source: Plant Direct. 2025 Feb 12;9(2):e70045. doi: 10.1002/pld3.70045 (PMC11815712; doi:10.1002/pld3.70045)
Supplement: Supplementary file 1 — Data S1 Supporting Information. [file PLD3-9-e70045-s001.pdf]

Plant Direct
Open Access

A promoter collection for cell-targeted analysis within the stomatal complex

Thanh-Hao Nguyen[1*], Jovaras Krasauskas[2,3*], Thu Binh-Anh Nguyen[1*], Azka Noureen[2*], Mark Smedley[2], John M. Christie[1], Wendy Harwood[2], Michael R. Blatt[1,4], Penny Hundleby[2]

Decision Letter Round 1:

December 22, 2024

Prof. Mike R. Blatt
University of Glasgow
Laboratory of Plant Physiology and Biophysics
Glasgow G12 8QQ
United Kingdom

RE: A promoter collection for cell-targeted analysis within the stomatal complex

Dear Dr. Blatt:

Thank you for submitting to Plant Direct. All required reviews have been returned and we have now finished our evaluation of your manuscript. In light of the reviewers' and editor's comments, further revisions are needed before the paper can be accepted for publication in Plant Direct.

Please view the editors' and reviewers' comments below and use their suggestions as a guide while you work on your revision.

When uploading the revised version of this article, please be sure to include the following:

-A word document that contains your response to the reviewers. You should respond to each reviewer comment and note the changes made to the manuscript. If you do not agree with a reviewer's comment and choose not to make a suggested revision, please explain why. Please try to provide as complete an answer as possible to each reviewer's criticisms.
-A tracked changes document with each change highlighted
- A clean version of the latest version of the manuscript

The manuscript presents a good description of an useful resource that will benefit many laboratories working in a variety of plants. In general, the paper is well-written and provides enough detail. I acted as reviewer in addition to being Editor to avoid delaying the review process any longer. I have only a couple of minor concerns: it was unclear to me the specific ends of each promoter sequence, since different authors use different criteria to determine them, and there is no common criteria described in the text. Is the 3'end of the promoters defined as the last nucleotide before transcript initiation (for the longest known 5'UTR)? Or is the size provided in Table 1 relative to the translation start codon?
I tried to look at the sequences in Addgene, and the codes provided in the table do not seem to work (this is my secind concern). I am not familiar with the Addgene submission process, so perhaps the

plasmids are not released until publication, please clarify.

To upload your revision, please click the link below.
https://plantdirect.msubmit.net/cgi-bin/main.plex?el=A4Lr6kl1A5ec4I3A9ftdd98e2pXGxHJPR2NIlNcQZ

In order to provide as timely a service as possible, we ask that your revision is resubmitted within three months after receipt of this request. If an extension is needed, please send a request, along with a brief explanation, to the editorial office at plantdirect@wiley.com .

Please note that, in addition to publishing reviewer comments, the author's responses to review comments will also be published alongside the final version of the paper. If you would not like the author's responses to be published, please contact the editorial office at plantdirect@wiley.com .

Thank you very much for giving us an opportunity to review your work. I look forward to receiving the next version.

Sincerely,

Gustavo MacIntosh

Editor, Plant Direct

--- Editor comments:
The manuscript presents a good description of an useful resource that will benefit many laboratories working in a variety of plants. In general, the paper is well-written and provides enough detail. I acted as reviewer in addition to being Editor to avoid delaying the review process any longer. I have only a couple of minor concerns: it was unclear to me the specific ends of each promoter sequence, since different authors use different criteria to determine them, and there is no common criteria described in the text. Is the 3'end of the promoters defined as the last nucleotide before transcript initiation (for the longest known 5'UTR)? Or is the size provided in Table 1 relative to the translation start codon?
I tried to look at the sequences in Addgene, and the codes provided in the table do not seem to work (this is my secind concern). I am not familiar with the Addgene submission process, so perhaps the plasmids are not released until publication, please clarify.

--- Reviewer comments:
Reviewer #1:

Stomatal movement is crucial for controlling gas exchange and transpiration, and the study of guard cell kinetics forms the foundation for improving stomata efficiency to balance water loss and CO_2 entry. Guard cell kinetics are influenced by the epidermal cells surrounding the guard cells. Nguyen et al. aimed to identify promoters specific to guard cells and surrounding epidermal cells across dicot and monocot species, to enable studies focusing on specific cell types. They generated plants transformed with selected promoters driving GUS reporters.

Guard cell specific promoters, pAtMYB60, pStKST1 and pAtGC1, were confirmed in Arabidopsis and Brassica but not in barley. pZmCST1 was specific to subsidiary cells in barley. However, no promoters were found to be specific to epidermal cells and excluded from guard cells in the two dicot species. Overall, the aim of the study is clear, and the methodology is solid. The images in the results section are of high quality. The conclusions can be more precise. For example, the only cell type specific promoter found in barley is pZmCST1 in subsidiary cells, highlighting some limitations for cell type specific studies.
I also have two additional minor suggestions:
1. The study focused on the epidermis. But I wonder if any of the promoter showed activity in mesophyll cells. This could provide insights for researchers studying epidermal cells in the leaf.
2. Table 1 summarizes the expression patterns of the promoters from the literature. Could a similar table be created to summarize the expression patterns observed in the three species studied here? This would help readers quickly extract useful information.

Decision Letter Round 2:

January 18, 2025
Prof. Mike R. Blatt
University of Glasgow
Laboratory of Plant Physiology and Biophysics
Glasgow G12 8QQ
United Kingdom

MSID: 2024-01621R1
MS TITLE: A promoter collection for cell-targeted analysis within the stomatal complex

Dear Dr. Mike Blatt:

I am pleased to inform you that your manuscript "A promoter collection for cell-targeted analysis within the stomatal complex" has been accepted for publication in Plant Direct.

Your article will appear online in the next available issue of Plant Direct. To ensure your article gets published as quickly as possible, please pay attention to the steps detailed below. We have found that most of the delays happen at this stage, especially at the payment stage, so please respond as quickly as possible when prompted.

License Agreement: Once your article has been accepted it will move to Production and undergo admin and file checking - you may receive an email with any queries we have at this stage. When all required items are received by the publisher and queries resolved, the corresponding author will receive an email from Wiley's Author Services system which will ask them to log on at https://authorservices.wiley.com/bauthor and will present them with the appropriate license for completion. Your article cannot be published until both the signed license agreement and payment of the article fee have been received.

Payment of the Open Access Article Publication Fee: All articles published in Plant Direct are fully open access: immediately and freely available to read, download and share. Plant Direct charges a publication

fee to cover the publication costs. The corresponding author for this manuscript should have already received a quote from the payments team (cs-openaccess@wiley.com) with the estimated article publication fee; please email cs-openaccess@wiley.com if this has not been received. The corresponding author should log on to the Wiley Author Services site, where the publication fee can be paid by credit card or an invoice. Pro Forma can also be requested. Payment of the publication charge must be received before the article will be published online.

Proofs: You will have the opportunity to look over your paper once more when you receive the author proofs for your article. The proofs will be with you in approximately two weeks. Please note that, in addition to publishing reviewer comments, the author's responses to review comments will also be published alongside the final version of the paper. If you would not like the author's responses to be published, please contact the editorial office at plantdirect@wiley.com .

Promotion of your article: You can help your research get the attention it deserves! Check out Wiley's free Promotion Guide for best-practice recommendations for promoting your work at www.wileyauthors.com/eeo/guide. And learn more about Wiley Editing Services which offers professional video, design, and writing services to create shareable video abstracts, infographics, conference posters, lay summaries, and research news stories for your research at www.wileyauthors.com/eeo/promotion.

Thank you again for your contribution to Plant Direct. If you have any questions, feel free to contact the editorial office at plantdirect@wiley.com .

Sincerely,

Gustavo MacIntosh

Editor, Plant Direct

Response to Reviewer:

15. January 2025

Gustavo Macintosh Editor, Plant Direct

Dear Gustavo,

Thank you for your efforts in handling this manuscript. We appreciate that you also took on the task of reviewing as well, given the (almost) three month delay incurred in handling.

In response to your queries, the positions of the promoter sequences relative to the start codon ATG are now clearly indicated in the new column of Table 1 under the column header 'Position from ATG'. As for the Addgene codes, these will not be functional until the manuscript has been accepted and has a DOI.

With regard to further changes to the manuscript, these are so minor - addition of the column to Table 1 (noted above), addition of Table 2 and its callouts - that I have not included a markup/track change version to the manuscript. I trust that this will not be a problem.

Yours sincerely,

Mike Blatt

Response to the Reviewers

Reviewer 1

We thank the reviewer for their supportive comments.

With regard to precision in the text, we cannot be more concise. For the avoidance of doubt, we now include Table 2, as suggested by the reviewer, which lists the promoters and the expression patterns we observed.

Additional minor suggestions

1. "I wonder if any of the promoter showed activity in mesophyll cells."

While we did not focus on expression in the leaf or other mesophyll tissues, it is clear from the survey images provided that sub-epidermal expression is minimal, if present. Where such expression is evident, it is clearly associated with the vascular tissues. We stated this point explicitly (cf. L. 138,145,158).

2. "Could a similar table be created to summarize the expression patterns observed in the three species studied here?"

Thank you for the suggestion. Table 2 now provides this information.
